# A Multi-Task EfficientNetV2S Approach with Hierarchical Hybrid Attention for MRI Enhancing Brain Tumor Segmentation and Classification

**DOI:** 10.3390/brainsci16010037

**Published:** 2025-12-27

**Authors:** Nawal Benzorgat, Kewen Xia, Mustapha Noure Eddine Benzorgat, Malek Nasser Ali Algabri

**Affiliations:** 1 School of Electronics and Information Engineering, Hebei University of Technology, Tianjin 300401, China; kwxia@hebut.edu.cn (K.X.); mbenzorgat97@gmail.com (M.N.E.B.); 2 Faculty of Computer and Information Technology, Sana’a University, Sana’a 37444, Yemen; malekye@su.edu.ye

**Keywords:** MRI brain tumor, segmentation and classification, multi-task learning, hierarchical hybrid attention, multi-scale feature fusion

## Abstract

**Background:** Brain tumors present a significant clinical problem due to high mortality and strong heterogeneity in size, shape, location, and tissue characteristics, complicating reliable MRI analysis. Existing automated methods are limited by non-selective skip connections that propagate noise, axis-separable attention modules that poorly integrate channel and spatial cues, shallow encoders with insufficiently discriminative features, and isolated optimization of segmentation or classification tasks. **Methods:** We propose a model using an EfficientNetV2S backbone with a Hierarchical Hybrid Attention (HHA) mechanism. The HHA couples a global-context pathway with a local-spatial pathway, employing a correlation-driven, per-pixel fusion gate to explicitly model interactions between them. Multi-scale dilated blocks are incorporated to enlarge the effective receptive field. The model is applied to a multiclass brain tumor MRI dataset, leveraging shared representation learning for joint segmentation and classification. **Results:** The design attains a Dice score of 92.25% and a Jaccard index of 86% for segmentation. For classification, it achieves an accuracy of 99.53%, with precision, recall, and F1 scores all close to 99%. These results indicate sharper tumor boundaries, stronger noise suppression in segmentation, and more robust discrimination in classification. **Conclusions:** The proposed framework effectively overcomes key limitations in brain tumor MRI analysis. The integrated HHA mechanism and shared representation learning yield superior segmentation quality with enhanced boundary delineation and noise suppression, alongside highly accurate tumor classification, demonstrating strong clinical utility.

## 1. Introduction

Brain tumors are serious neurologic diseases characterized by abnormal cell growth in the brain, leading to a diverse range of symptoms including seizures, headaches, and cognitive or motor deficits [1,2]. The clinical behavior of these tumors is highly variable and difficult to predict, as their size, location, and growth patterns vary widely, complicating diagnosis and treatment planning [3]. The significant burden of brain tumors is underscored by recent statistics; for instance, in 2023, an estimated 24,810 new cases of brain cancer and 18,990 related deaths were reported in the United States, with higher tumor grades generally correlating with more aggressive disease and poorer outcomes [4]. Magnetic resonance imaging (MRI) has emerged as the primary non-invasive tool for brain tumor assessment due to its high soft tissue contrast and fine spatial resolution, which are essential for delineating a tumor’s size, shape, and location [5]. This imaging is critical for evaluating diverse tumor types. For example, meningiomas, which account for a large share of surgical cases, can range from benign to aggressive forms that remain asymptomatic until compressing nearby structures [6]. Similarly, pituitary tumors often present with endocrine disturbances or mass effect on adjacent regions like the optic chiasm [7]. While MRI is indispensable, the definitive diagnosis still often requires biopsy or surgery.

Manual interpretation of MRI is subjective, time-consuming, and prone to error [8]. Heterogeneity in tumor type and imaging appearance makes manual segmentation difficult and typically requires expert neuroradiologists, which motivates automated approaches based on image processing and deep learning. These automated segmentation and classification methods can assist with accurate estimation of tumor volume, shape, and boundaries and can support diagnostic and therapeutic decisions. However, many existing systems are designed for a single task only, which limits practical value when both classification and segmentation are required in the same workflow. Foundational architectures such as U-Net face important constraints in this setting. Standard skip connections transfer encoder features directly to the decoder without selection, which propagates noise and irrelevant background and blurs lesion boundaries. Attention mechanisms can reduce these effects, but common designs process channel cues and spatial cues separately and do not model their interaction. The problem becomes more pronounced when encoders have limited capacity to extract strong and discriminative representations from small, low-contrast, or complex tumor patterns. Systems that optimize only one task also miss potential gains from shared representation learning.

To address these challenges, we propose a multi-task learning framework built on an EfficientNetV2S backbone with a novel Hierarchical Hybrid Attention (HHA) mechanism that unifies brain tumor segmentation and classification in a single network. Unlike existing axis-separable attention modules, our HHA jointly models global context and local spatial details through a correlation-driven adaptive fusion strategy. The core innovation lies in transforming skip connections from passive feature conduits into selective regulators that dynamically filter noise while preserving tumor-relevant structures. This is complemented by a multi-scale feature fusion strategy and an optional Enhanced Hybrid Attention (EHA) variant that incorporates self-attention for capturing long-range dependencies.

The framework is further enhanced through a multi-contrast preprocessing approach that provides complementary intensity perspectives for robust feature learning. By coupling an advanced encoder with task-specific decoders, our model overcomes key limitations of prior work, including weak encoders, naive skip connections, and isolated channel-spatial attention designs. The main contributions of this work are summarized as follows:

1. A novel Hierarchical Hybrid Attention (HHA) mechanism that integrates global-context and local-spatial pathways with correlation-driven, per-pixel fusion, enabling joint modeling of channel- and spatial cues.
2. A unified multi-task learning framework that simultaneously performs tumor segmentation and classification using a shared encoder, eliminating the need for separate models.
3. The model leverages an EfficientNetV2S backbone combined with HHA, replacing standard U-Net weak encoder and simple feature concatenation. This advanced backbone with dynamic resizing handles variations in tumor appearance, size, and location, ensuring robust performance across diverse MRI scans.
4. Enhanced Hybrid Attention (EHA) variant that incorporates multi-head self-attention to capture long-range dependencies, further improving contextual modeling.
5. Multi-contrast preprocessing strategy that enriches input representation through complementary contrast enhancement, enabling the model to learn complementary features across contrast scales.
6. Comprehensive experimental validation demonstrating state-of-the-art performance on a public brain tumor MRI dataset, with superior results in both segmentation (92.25% Dice, 86% Jaccard) and classification (99.53% accuracy).

## 2. Related Work

Brain tumor segmentation and classification have advanced with deep learning, which addresses the limits of classical image processing. Early studies used threshold-based segmentation, edge detection, and clustering that depended on extensive feature engineering. Taheri et al. [9] applied level set segmentation with threshold-driven speed functions, and Islam et al. [10] used superpixel generation with K means clustering. These methods struggled with tumor heterogeneity, low-contrast imaging, and complex boundaries, which motivated more capable models.

The U-Net architecture by Ronneberger et al. [11] transformed medical image segmentation through a symmetric encoder–decoder design with skip connections. Many extensions targeted specific gaps. Oktay et al. [12] proposed Attention U-Net, where attention gates select informative features passed through skip connections and improve localization for multi organ tasks. Zhang et al. [13] added attention gates to Residual U-Net for automatic MRI brain tumor segmentation. Alom et al. [14] introduced Recurrent Residual U-Net to refine feature extraction and improve efficiency. Diakogiannis et al. [15] presented ResUNet for semantic segmentation in remote sensing, later adapted to medical imaging. Zhao and Jia [16] designed a multi-scale CNN to handle variable tumor size with improved scale consistency, and Kamnitsas et al. [17] proposed DeepMedic, a dual path three-dimensional CNN for brain lesion segmentation.

Multi-task learning has highlighted the synergy between segmentation and classification. Rabby et al. [18] presented BT Net, a VGG16-based multi-task architecture combined with a U-Net variant that performs segmentation, classification, and localization on MRI, reaching 97% classification accuracy and a Dice similarity of 0.86. Kordnoori et al. [19] developed a deep multi-task model with a shared encoder, a decoder for segmentation, and a multilayer perceptron for classification of three common primary brain tumors, achieving 97% accuracy for both tasks. Hussain et al. [20] introduced Residual Attention U-Net for joint learning, reporting 89.30% Jaccard similarity, 91.10% Dice coefficient, and 93.35% segmentation accuracy. EfficientNet families have gained traction due to compound scaling that balances depth, width, and input resolution. Preetha et al. [21] combined multi-scale Attention U-Net with an efficientNetB4 encoder for brain tumor segmentation and reported 99.79% accuracy, a Dice coefficient of 0.9339, and an intersection over union of 0.8795, identifying B4 as an effective compromise between accuracy and computational cost.

Despite progress, several limitations remain, such as naive skip connections, weak encoders, and the isolation of channel attention and spatial attention while adopting attention designs (Table 1). Finally, many systems address only segmentation or only classification, which reduces the benefit of shared information between complementary tasks.

## 3. Methodology

### 3.1. Framework Overview

**Figure 1:**
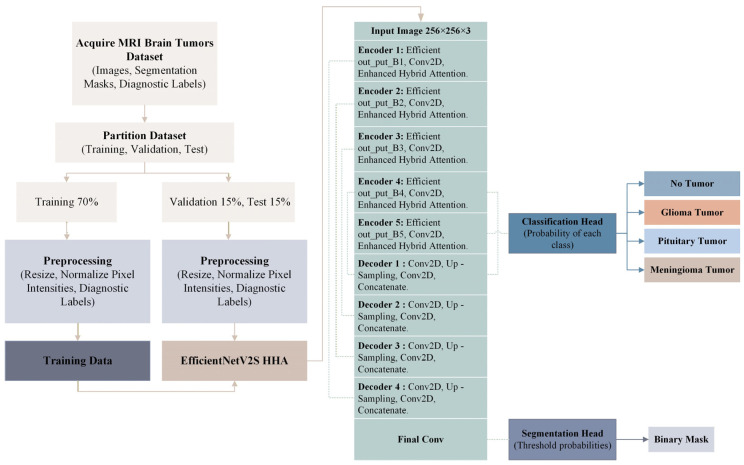
Detailed architecture of the proposed EfficientNetV2S HHA model.

To provide a clear view of the overall design, Figure 1 summarizes the complete workflow of the proposed framework. The pipeline begins with data acquisition and preprocessing, then proceeds to training and inference using an EfficientNetV2S backbone integrated with the Hierarchical Hybrid Attention (HHA) mechanism. The framework performs segmentation and classification in a unified manner, enabling simultaneous tumor delineation and type recognition within a single computational flow.

### 3.2. Data Pre-Processing

**Figure 2:**
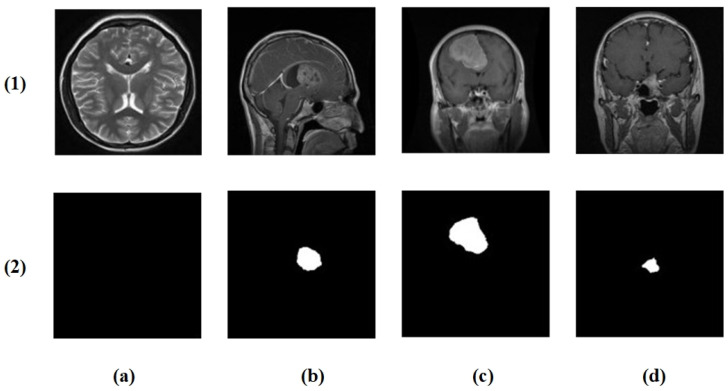
Example cases from the brain tumor segmentation dataset: (**1**) original MRI slices and (**2**) corresponding ground truth masks. Columns (**a**–**d**) show the following four classes: no tumor, glioma, meningioma, and pituitary tumor.

The brain tumor segmentation dataset from Kaggle [22] comprises 4237 T1-weighted contrast-enhanced MRI images (512 × 512 pixels) across the following four classes: no tumor (1595), glioma (650), meningioma (999), and pituitary tumor (994), with corresponding ground truth masks (Figure 2).

**Figure 3:**
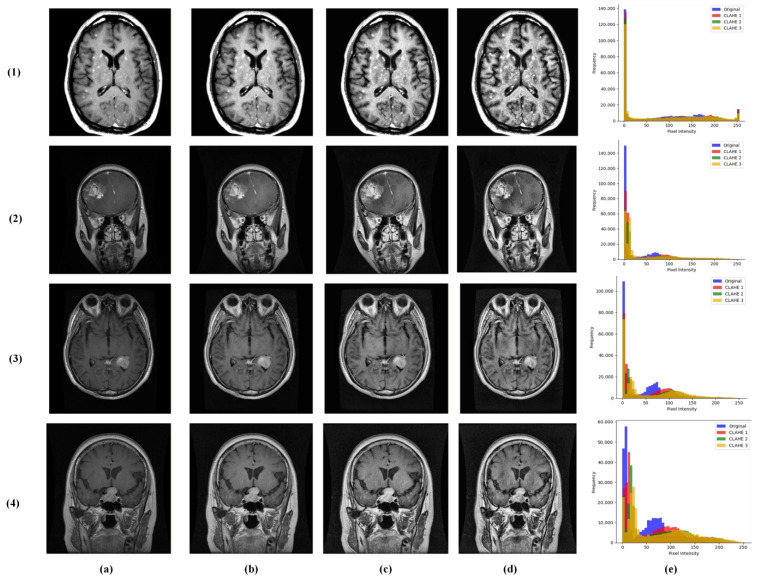
Visualization of multi-contrast CLAHE preprocessing results across the following brain tumor classes: (**1**) no tumor, (**2**) glioma, (**3**) meningioma, and (**4**) pituitary tumor. Columns represent (**a**) original input MRI, (**b**) conservative CLAHE, (**c**) moderate CLAHE, (**d**) aggressive CLAHE, and (**e**) histogram.

We introduced a multi-contrast CLAHE enhancement strategy implemented using the OpenCV library [23]. Each grayscale MRI was processed through the following three CLAHE configurations: conservative (clipLimit = 1.0, grid = 4 × 4), moderate (clipLimit = 2.0, grid = 8 × 8), and aggressive (clipLimit = 3.0, grid = 12 × 12). These were stacked as separate channels, providing the model with complementary contrast perspectives for robust feature learning (Figure 3).

Multi-contrast images were resized to 256 × 256 pixels (bilinear interpolation) and normalized, while masks used the nearest-neighbor interpolation. The dataset was divided at the patient level into 70% training, 10% validation, and 20% testing sets, maintaining class distribution. This comprehensive annotation supports integrated multi-task learning for simultaneous tumor classification and segmentation.

### 3.3. Proposed EfficientNetV2S HHA Architecture

**Figure 4:**
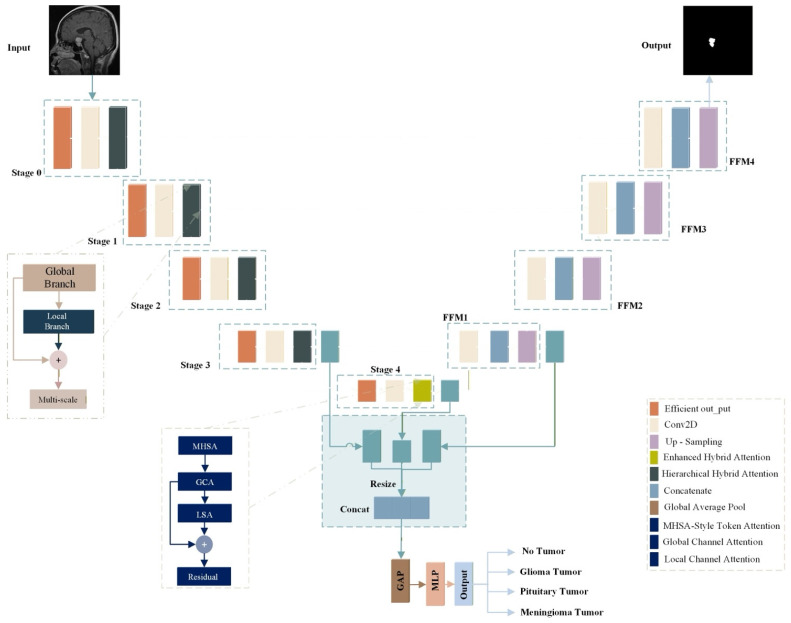
Overview of the proposed multi-task brain tumor model.

The proposed EfficientNetV2S HHA performs brain tumor segmentation and classification at the same time using a shared encoder with two task specific branches. Figure 4 outlines the design. The segmentation branch follows a U-shaped encoder–decoder layout with hierarchical stages that support multi-scale feature extraction and fusion. The classification branch draws features from several encoder depths to distinguish glioma, meningioma, pituitary tumor, and no tumor.

An EfficientNetV2S encoder [24], loaded with pre-trained ImageNet weights via TensorFlow’s Keras API [25], forms the core feature extractor and provides a stronger representational capacity than standard U-Net encoders. Instead of naive skip connections that pass features without selection, each encoder level integrates an HHA module that filters and enhances information before fusion.

The overall scheme is asymmetric across tasks. High-level semantic features are routed to the classification head, while the full hierarchy of features is used for precise boundary estimation in the segmentation path. This integrated design allows a single model to deliver both tasks, avoiding separate networks and addressing the limits of single task systems.

### 3.4. Generalized Hierarchical Hybrid Attention (HHA)

To refine information flow along skip connections, we design HHA as a unified operator with the following two coordinated branches: a global-context path and a local-spatial path. These are fused via a correlation-driven, per-pixel gate, followed by multi-scale enhancement and residual gating. HHA jointly processes global and local evidence and learns their interaction prior to fusion. Specifically, we derive a cross-branch correlation map between globally and locally enhanced features, and a two-channel fusion gate outputs per-pixel mixing weights for the two paths. A 1 × 1 → 3 × 3 → dilated 3 × 3 block expands the receptive field, and a residual gate adaptively blends refined and original features. For the global-context path:

(1) $$G\left(X\right)=\sigma \left(W_{g}^{\left(2\right)}\delta \left(W_{g}^{\left(1\right)}GAP\left(X\right)\right)\right),\;X_{g}=X⊙G\left(X\right)$$

where GAP is global average pooling, δ is ReLU, σ is sigmoid, and $W_{g}^{\left(1\right)}$ and $W_{g}^{\left(2\right)}$ are 1×1 convolutions. Speaking of the local-spatial path:

(2) $$L_{1}=\phi_{1}\left(X\right),\;L_{2}=\phi_{2}\left(X\right);\;A_{L}=\sigma \left({\mathrm{Avg}}_{c}\left(L_{1}\right)+{\mathrm{Max}}_{c}\left(L_{2}\right)\right),\;X_{l}=X⊙A_{L}$$

where $\phi_{1,2}$ are 3×3 conv-GroupNorm-ReLU-Dropout stacks, and ${Avg}_{c},\;{Max}_{c}$ denote averaging and max-pooling across the channel dimension, respectively. Also, for cross-branch correlation, we have:

(3) $$C_{corr}=\sigma \left({\mathrm{Avg}}_{c}\left(X_{g}⊙X_{l}\right)\right)$$

where $X_{g}$, $X_{l}$ represent the globally enhanced and locally enhanced versions of the same input X.

Unlike the Convolutional Block Attention Module (CBAM), which applies sequential, axis-separable attention on a channel stage and a spatial stage, HHA addresses limitations of CBAM in three ways as follows: (i) it uses learned local branches alongside a global-context path; (ii) it computes a cross-branch correlation map and per-pixel, two-channel fusion weights prior to fusion; and (iii) it applies multi-scale enhancement and residual gating to refine outputs. We further introduce an Enhanced Hybrid Attention (EHA) variant that inserts multi-head self-attention to capture long-range dependencies before channel- and spatial gating. This correlation-driven, joint design turns skip connections from passive conduits into selective regulators.

#### 3.4.1. Enhanced Hybrid Attention (EHA)

While HHA focuses on coupling global and local cues through correlation-driven fusion, the Enhanced Hybrid Attention (EHA) variant extends this design by embedding a multi-head self-attention (MHSA) mechanism to capture long-range dependencies and contextual consistency across the entire feature map. Functionally, EHA begins by projecting the input feature map into query (Q), key (K), and value (V) tensors through 1 × 1 convolutions. The MHSA operation then computes pairwise dependencies between all spatial positions, allowing each pixel to aggregate information from distant regions, an ability absent in traditional convolutional or CBAM-like attention mechanisms. The attention-weighted features are reshaped back to spatial form and subsequently refined through channel and spatial gating modules. By combining MHSA with hierarchical gating and multi-scale convolutional fusion, EHA provides global context awareness and local feature precision simultaneously. This hybrid structure unifies transformer-style global reasoning with convolutional spatial selectivity, offering a stronger contextual foundation for both the segmentation decoder and the classification head. Concretely, for $X\in R^{H\times W\times C}$ with h heads and head size $d_{h}$:

(4) $$Q=W_{Q}*X,\;K=W_{K}*X,\;V=W_{V}*X$$

For each head i:

(5) $${Attn}_{i}=\mathrm{Softmax}\left(\frac{Q_{i}K_{i}^{⊤}}{\sqrt{d_{h}}}\right)V_{i},\;X_{a}={\mathrm{Concat}}_{i}\left({Attn}_{i}\right)$$

#### 3.4.2. Correlation-Driven Fusion Gate

The outputs from the global-context and local-spatial attention pathways are integrated through a sophisticated fusion mechanism that forms the core of our HHA approach. This integration employs multiple complementary strategies to ensure optimal feature refinement and information preservation.

The fusion process begins with cross-pathway correlation analysis, where the interaction between globally and locally enhanced features is quantified:

(6) $$F(X)=\sigma (\varphi (X))\in [0,1{]}^{H\times W\times 2}\;$$

where φ represents a convolutional layer producing a two-channel output.

With channels α, β, the integrated feature representation is computed through a multi-component fusion strategy:

(7) $$F_{\mathrm{fuse}\;}=\alpha X_{g}+\beta X_{l}+C_{\mathrm{corr}\;}\left(X_{g}+X_{l}\right)$$

This triple-fusion approach ensures that both individual pathway contributions and their synergistic interactions are preserved. The framework further enhances the integrated features through a multi-scale convolutional block employing sequential operations.

A gated residual connection finally refines the output through adaptive feature preservation:

(8) $$Y=\psi \left(F_{\mathrm{fuse}}\right)$$

(9) $$R=\sigma \left(\mathrm{GAP}\left(Y\right)-\mathrm{GAP}\left(X\right)\right)$$

(10) $$Z=R⊙Y+\left(1-R\right)⊙X$$

This intelligent integration mechanism enables the network to automatically learn the optimal balance between global-contextual understanding and local-spatial details, while adaptively preserving or transforming features based on their informational value. The hierarchical approach ensures robust feature enhancement across diverse tumor manifestations and imaging conditions.

### 3.5. Multi-Scale Feature Fusion

We perform progressive multi-scale fusion on HHA-refined features from the EfficientNetV2S [24] pyramid. After each encoder stage, HHA produces selectively filtered maps; these maps are then aligned (Resize-to-Match) and fused top-down (FFM1→FFM4), propagating hierarchically modulated attention into the decoder.

As shown in Figure 4, the feature maps from different backbone stages are first processed through the hybrid attention modules to enhance relevant features and suppress noise. The higher-level features (from deeper stages) are then upsampled and resized to match the spatial dimensions of lower-level features using Resize-to-Match layers. This ensures that features from different scales are properly aligned before fusion.

The feature fusion process follows a hierarchical approach where first, Stage 4 features are upsampled and combined with Stage 3 features to form feature fusion module 1 (FFM1). Then, FFM1 is further upsampled and combined with Stage 2 features to form FFM2. Next, FFM2 is upsampled and combined with Stage 1 features to form FFM3. Finally, FFM3 is upsampled and combined with Stage 0 features to form FFM4.

This progressive feature fusion ensures that high-level semantic information from deeper layers is effectively combined with low-level spatial details from shallower layers, creating a comprehensive representation for both classification and segmentation tasks.

#### 3.5.1. Classification Head

The classification head uses high-level semantic features to determine tumor type. It operates on the attention-refined outputs from Stage 3 (F_l3), Stage 4 (F_l4), and the first fusion module (F_ffm1). Each input first passes through its hybrid attention module to obtain a refined feature set. To enable fusion, features with a larger spatial stride are upsampled and resized to match the spatial size of the Stage 3 features. The three processed tensors are then concatenated to form a single representation. Global average pooling converts this map into a fixed-length vector, which is fed to fully connected layers that generate the final class prediction. This design emphasizes the most discriminative cues for recognition while preserving computational efficiency.

#### 3.5.2. Segmentation Head

The segmentation head uses the complete feature pyramid to refine boundaries by operating on the final fused-feature map FFM4. The input passes through a stack of convolutional blocks, where each block applies group normalization, a ReLU activation, and a 3×3 convolution. The resulting map is then upsampled to 256 × 256 pixels. A 1×1 convolution produces per-pixel class scores, and a sigmoid activation generates the binary mask. This design preserves detailed spatial cues gathered across scales while keeping the computation efficient.

### 3.6. Multi-Task Loss Function

The model is optimized using a multi-task loss function that combines objectives for both classification and segmentation. For the classification task, which trains the model to classify the whole image, the standard cross-entropy loss is employed, defined as:

(11) $$L_{cls}=-\frac{1}{N}\sum_{k=1}^{N}\;\sum_{c=1}^{C}\;y_{kc}\log\left(p_{kc}\right)$$

where $y_{kc\;}$ is the ground truth label, $p_{kc}$ is the predicted probability for sample k and class c, N is the batch size, and C is the number of classes.

For the segmentation task, a combination of losses is used to address the challenge of class imbalance and to refine structural details. The primary component is the Dice loss,

(12) $$L_{\mathrm{dice}\;}=1-\frac{2\sum_{i}\;p_{i}g_{i}}{\sum_{i}\;p_{i}+\sum_{i}\;g_{i}}\;$$

where $p_{i}$ and $g_{i}$ denote the predicted segmentation map and the ground truth, respectively. 

### 3.7. Hyperparameters Setting

The proposed hybrid multi-task framework was trained on 256 × 256 × 3 MRI patches using a batch size of 16 over 150 epochs, with the Adam optimizer and an initial learning rate of 1 × 10^−4^. The model was optimized using the following combined loss function: sparse categorical cross-entropy for classification and a custom Dice loss for segmentation, with dynamic task weighting via an automatic weighted loss (AWL) layer. Training was monitored using the following epoch-wise metrics: Dice coefficient, Jaccard index, classification accuracy, segmentation accuracy, precision, recall, and F1 score. Early stopping was applied with a patience of 5 epochs based on validation loss, and ReduceLROnPlateau reduced the learning rate by a factor of 0.2 when validation loss plateaued for 5 consecutive epochs. The best model weights were saved according to the lowest validation loss. All hyperparameters, including input size, batch size, number of epochs, optimizer, learning rate, attention module configurations, and fusion strategies, were fixed as defined in the model architecture and training script as summarized in Table 2. Algorithm 1 summarizes the proposed EfficientNetV2S HHA architecture’s steps.
**Algorithm 1.** EfficientNetV2S HHA algorithmAlgorithm of Multi-Task Brain Tumor Model Construction EfficientNetV2S HHASEQUENCE Input: MRI slices (images), binary masks, multi-class labels Output: Predicted brain tumor type and segmentation mask BEGIN 1.   Define EfficientNetV2S encoder as the backbone. 2.   Initialize Hierarchical Hybrid Attention (HHA) module. 4.   Preprocess images and masks (resize to 256 × 256, multi-contrast CLAHE, and normalize images to [0,1]). 5.    Extract multi-scale feature maps F0, F1, F2, F3, F4 from the encoder. 6.    Apply HHA on each feature map to obtain attentive skips S0…S4. 7.    Build top-down decoder with progressive fusion:   7.1 U4 = Concat(Up(S4), S3) → ConvBlock   7.2 U3 = Concat(Up(U4), S2) → ConvBlock   7.3 U2 = Concat(Up(U3), S1) → ConvBlock   7.4 U1 = Concat(Up(U2), S0) → ConvBlock 8.    Segmentation head:   8.1 SegFeat = Up(U1) → Conv1 × 1   8.2 Mask prediction: M_hat = Sigmoid (SegFeat) 9.    Classification head:   9.1 Z = Concat(S3, Resize(S4), Up(U4)) → ConvBlock   9.2 p = GAP → Dense → Dropout → Dense → Softmax 10.  Define task losses:   10.1 L_seg = 1 − Dice (M, M_hat)   10.2 L_cls = CrossEntropy(y, p)   10.3 L_total = w_seg · L_seg + w_cls · L_cls (automatic weighted loss) 11.  Compile model:   11.1 Optimizer = Adam (learning rate = 1 × 10^−4^)   11.2 Metrics: Dice, Jaccard (IoU), accuracy, precision, recall, F1 12.  Train:   12.1 For each epoch:      (a) Forward pass → compute L_total      (b) Backpropagate and update backbone, HHA, heads, and AWL weights      (c) Validate on hold-out set, apply LR scheduler and early stopping 13.  Inference:   13.1 Given a test image I: (M_hat, p) = Model(I)   13.2 Threshold M_hat > τ (e.g., τ = 0.5) to obtain binary mask   13.3 Predicted class: y_hat = argmax(p) END END SEQUENCE

### 3.8. Experimental Environment

All experiments were conducted on Kaggle’s cloud-based computing environment, designed to support intensive data processing and deep learning workflows. The setup offered 73.1 GB of persistent storage to accommodate raw datasets, trained model weights, and intermediate outputs. With 13 GB of RAM, data pre-processing and in-memory operations were performed smoothly, ensuring minimal bottlenecks during pipeline execution. All training and evaluation runs were performed on an identical hardware/software configuration to guarantee fair comparisons. The computational instance provided two NVIDIA Tesla T4 GPUs (each with 15.9 GB of VRAM; training used a single GPU for consistency) and 4 Intel Xeon CPU cores. The software stack included CUDA 12.5.1, cuDNN 9, TensorFlow 2.18.0 with Keras 3.8.0, and Python 3.11.13. This dedicated GPU accelerated the training of all neural networks, including the proposed architecture and every baseline model in the ablation study (Table 6). Furthermore, an additional 19.5 GB of output storage was allocated to preserve final predictions, model diagnostics, and graphical analyses generated throughout the experimentation phase.

### 3.9. Evaluation Metrics

The model performance was evaluated using a comprehensive set of metrics to assess segmentation quality and classification accuracy. The Dice coefficient was calculated to quantify the overlap between predicted and ground truth segmentations, defined as:

(13) $$\mathrm{Dice}\;=\frac{2TP}{2TP+FP+FN}$$

where TP, FP, and FN represent true positives, false positives, and false negatives, respectively.

In addition, the Jaccard index (IoU) provided an independent assessment of spatial precision by evaluating the ratio of correctly identified regions to their union with the ground truth:

(14) $$IoU=\frac{TP}{TP+FP+FN}$$

Classification reliability was further characterized through precision, which reflects the proportion of accurate positive predictions among all predicted positives:

(15) $$\mathrm{Precision}\;=\frac{TP}{TP+FP}$$

Sensitivity, which measures the model’s ability to correctly identify all actual positive instances, was calculated using the formula:

(16) $$\mathrm{Sensitivity}\;=\frac{TP}{TP+FN}$$

The harmonic mean of precision and recall, the F1 score, delivered a balanced metric for overall classification efficacy.

(17) $$F_{1}-Score=\frac{2\times TP}{2\times TP+FP+FN}\;$$

Additionally, the overall accuracy was computed to assess global classification correctness, though its interpretation requires caution in imbalanced datasets.

(18) $$\mathrm{Accuracy}\;\left(ACC\right)=\frac{TP+TN}{TP+FN+TN+FP}$$

## 4. Result Analysis and Discussion

### 4.1. Performance Evaluation of the EfficientnetV2S HHA

The proposed EfficientnetV2S HHA demonstrated exceptional performance across both segmentation and classification tasks. As summarized in Table 3, the model achieved a Dice coefficient of 92.25% and Jaccard index of 85.77% for segmentation tasks, while attaining an overall classification accuracy of 99.53%. The precision, recall, and F1 score metrics all approached 99%, indicating robust and balanced performance across both tasks. The training accuracy curve demonstrates rapid convergence and stable performance throughout the training process, with the model achieving over 99% accuracy on the validation set and maintaining consistent performance through the remaining epochs.

The classification performance across individual tumor classes, as detailed in the classification report (Table 4), reveals near-perfect detection capabilities. The model achieved 100% precision and recall for the no tumor class, demonstrating exceptional specificity in identifying healthy cases. For pathological cases, glioma tumors were detected with 98% precision and 99% recall, while meningioma and pituitary tumors both achieved 99% precision and recall rates. This consistent high performance across all classes underscores the model’s robustness in handling the inherent class imbalance present in medical datasets.

**Figure 5:**
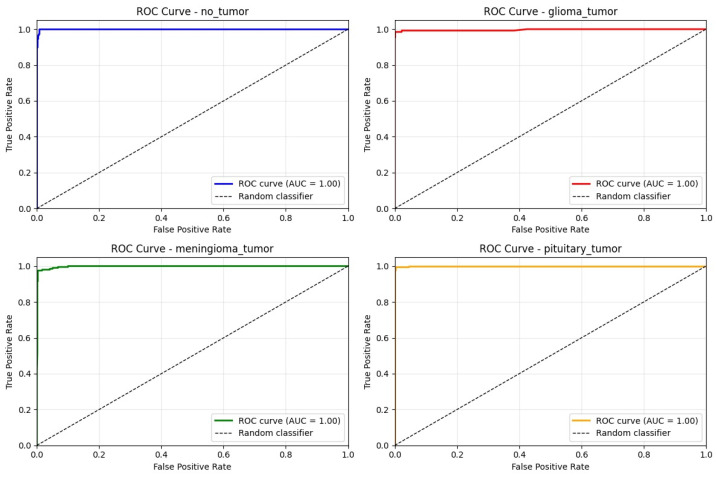
The ROC curve for each class on test set.

The receiver operating characteristic (ROC) analysis revealed area under the curve (AUC) values exceeding 99.80% for all tumor classes, confirming the model’s excellent discriminative capability and diagnostic reliability across the entire multi-class spectrum, as shown in Figure 5.

### 4.2. Data Splitting Strategy Analysis

To determine the optimal data partitioning strategy, four different train-validation-test splits were evaluated, as shown in Table 5. The 70-10-20 split configuration yielded the best overall performance, with a Dice coefficient of 92.25% and classification accuracy of 99.53%. This split provided sufficient training data (70%) while maintaining adequate validation (10%) and testing (20%) sets for robust evaluation. The 80-10-10 split showed competitive segmentation performance (91.99% Dice) but slightly lower classification accuracy (98.82%), suggesting potential overfitting with larger training proportions. The 60-20-20 split demonstrated the lowest performance across all metrics, indicating that reducing training data below 70% adversely affects model learning capability.

### 4.3. Ablation Study and Component Analysis

The ablation results in Table 6 clarify the role of each component in the framework. Beginning with the plain U-Net baseline, we obtain strong overlap and classification metrics (Dice 89.63%, Jaccard 82.67%, accuracy 98.58%, precision 98.61%, recall 98.47%, and F1 score 98.58%). Swapping the encoder for EfficientNetV2S slightly improves spatial overlap but lowers global classification aggregates (Dice 90.13% and Jaccard 83.56%; accuracy 96.35%, precision 96.40%, recall 96.32%, and F1 score 96.36%), indicating that stronger feature extraction alone does not guarantee better decision reliability. Adding the proposed Hierarchical Hybrid Attention (HHA) on top of efficientnetv2s yields consistent gains across all measures as follows: Dice rises to 92.25% and Jaccard to 85.77%, which are improvements of 2.12 and 2.21 points over efficientnetv2s, respectively; accuracy, precision, recall, and F1 score reach 99.53%, 99.53%, 99.48%, and 99.53%, representing increases of 3.18, 3.13, 3.16, and 3.17 points over efficientnetv2s. Relative to the U-Net baseline, HHA delivers +2.62 Dice, +3.10 Jaccard, and modest but consistent gains in classification metrics (+0.95 accuracy, +0.92 precision, +1.01 recall, and +0.95 F1 score). These patterns suggest that efficientnetv2s provides broader and more discriminative receptive fields, while HHA converts skip-connection fusion into a selective, correlation-aware process that sharpens boundaries and suppresses background responses, thereby improving both spatial overlap and final decision consistency.

### 4.4. Comparative Analysis with SOTA Methods

#### 4.4.1. Segmentation Performance Comparison

As shown in Table 7, our proposed EfficientnetV2S HHA model demonstrates competitive performance against contemporary segmentation approaches. Our model achieves a more balanced performance across both segmentation and classification tasks. Compared to traditional U-Net architectures (Ronneberger et al.: 83.45% Dice, 88.03% accuracy), our approach shows substantial improvements of approximately 10.67% in Dice coefficient and 13.24% in accuracy. The model also outperforms several recent approaches including El-Shafai et al. (2022) [26] and Sobhaninia et al. (2020) [27] across all evaluation metrics.

#### 4.4.2. Classification Performance Comparison

In classification tasks, as detailed in Table 8, our model achieves state-of-the-art performance with 99.53% accuracy, surpassing most existing methods including Ravinder et al. (95.01%), Cardoso et al. (92.00%), Ardan et al. (95.00%), and Ullah et al. (95.42%). The model demonstrates comparable performance to SYED et al. (99.40%) while providing the additional capability of simultaneous segmentation, which most classification-focused approaches lack.

#### 4.4.3. Comparison with the SOTA Architectures

The comparative evaluation across representative segmentation architectures confirms the advantage of the proposed design under a unified training protocol with both segmentation and classification heads. As shown in Table 9, U-Net attains 89.63% Dice and 98.58% accuracy, while U-Net3+ modestly improves boundary consistency (90.13% dice). Architectures with explicit attention perform better: Attention U-Net reaches 89.91% Dice, 83.33% Jaccard, and 98.41% accuracy. Transformer-style Swin-Unet (87.32% dice) and residual recurrent R2U-Net (77.97% Dice) trail on overlap metrics in this data regime, indicating sensitivity to training scale and optimization dynamics. ResUNet-a yields balanced but lower scores (88.21% Dice; 96.81% accuracy), reflecting its heavier regularization and design biases toward shape priors.

**Figure 6:**
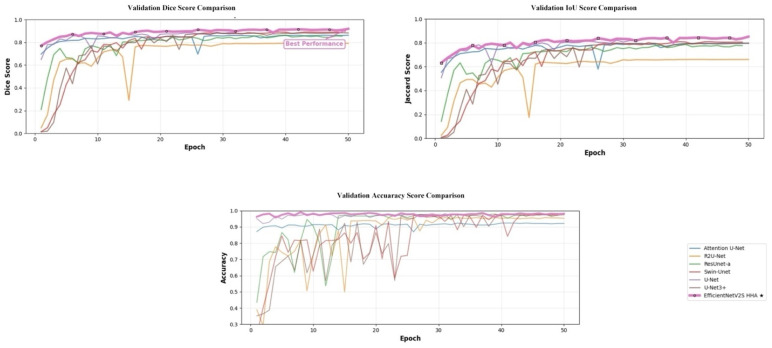
Validation learning curves (Dice, Jaccard, and accuracy) comparing SOTA baselines with the proposed EfficientNetV2S-HHA. The star denotes the model with the highest performance.

By contrast, the proposed EfficientNetV2S HHA attains 92.25% Dice, 85.77% Jaccard, and 99.53% accuracy, establishing the best results across all reported metrics. Relative to Attention U-Net, Dice and Jaccard rise by +0.34 and +0.44 percentage points, respectively, with a concurrent +0.12 point gain in accuracy; compared with the canonical U-Net, the improvements are +2.62 Dice, +3.10 Jaccard, and +0.95 accuracy. Precision, recall, and F1 score also peak at 99.53%, 99.48%, and 99.53%, surpassing the strongest baseline (99.41%, 99.30%, and 99.41%). These consistent deltas indicate that HHA’s correlation-guided fusion sharpens boundary localization without sacrificing image-level discrimination, while the EfficientNetV2S encoder supplies scale-balanced features that remain robust under the dual-task objective. Figure 6 represents training curves of each architecture.

The statistical significance of these improvements is further validated through multiple independent runs. Table 10 presents performance across different random seeds, revealing consistent results with low variance (Dice: 91.69% ± 0.43%). This confirms that the observed improvements over baseline architectures (e.g., +2.06% Dice over U-Net) are statistically robust and reproducible.

#### 4.4.4. Comparison with the SOTA Attentions

To further validate the efficiency and adaptability of the proposed EfficientNetV2S HHA model, we conducted a comparative analysis against several state-of-the-art attention mechanisms, including dual spatial attention (DSA), efficient channel attention (ECA), and convolutional block attention module (CBAM). Each attention module was independently integrated into the same architectural position as the proposed HHA mechanism, ensuring a fair and consistent evaluation setting. All other hyperparameters, training configurations, and data splits were preserved across experiments to isolate the contribution of the attention design itself.

As summarized in Table 11, the proposed HHA module consistently outperformed all compared attention variants in both segmentation and classification performance. The CBAM configuration achieved strong overall accuracy (98.41%) and balanced precision-recall behavior, reflecting its dual-attention efficiency. However, its spatial recalibration proved less effective in capturing deep contextual cues from heterogeneous tumor textures. The ECA variant provided notable gains over the baseline EfficientNetV2S (an increase of 1.28% Dice and 2.71% accuracy), validating the importance of refined channel weighting, though its lack of spatial adaptivity limited further gains in boundary precision. The DSA configuration demonstrated improved spatial discrimination with a Dice coefficient of 89.98% and classification accuracy of 97.44%, emphasizing the value of fine-grained feature localization.

By contrast, the proposed HHA module integrated global and local dependencies through a hybrid fusion process that dynamically balances semantic richness and boundary accuracy. This synergy yielded the highest Dice coefficient (92.25%) and classification accuracy (99.53%), marking relative improvements of 2.27%, 1.86%, and 3.12% over CBAM, DSA, and ECA configurations, respectively. The visual segmentation maps further illustrate HHA’s superiority, particularly in challenging tumor margins and low-contrast regions, where competing attentions either under-segmented or blurred structural boundaries.

The consistent improvements obtained by HHA confirm the advantage of combining multi-scale spatial refinement with global feature aggregation. Unlike prior single-dimension attentions, HHA adaptively learns context-aware importance weights that reinforce both regional coherence and discriminative strength. These findings establish HHA not merely as an incremental extension but as a unified attention formulation capable of surpassing established SOTA designs under identical experimental conditions.

#### 4.4.5. Evaluation of Segmentation and Classification Configurations

To further investigate the contribution of the dual-branch design in the proposed EfficientNetV2S HHA framework, the following two additional configurations were evaluated: a segmentation-only model and a classification-only model. Both variants retained the same encoder structure and training parameters, differing only in the removal of one task-specific branch. This analysis isolates the effect of joint optimization and shared feature learning between the two tasks.

As presented in Table 12, the segmentation-only configuration achieved a Dice coefficient of 91.13% and Jaccard index of 86.04%, confirming its strong capability for structural localization even without auxiliary supervision from the classification head. The classification-only model attained 98.02% accuracy and 98.23% F1 score, demonstrating robust discriminative power when trained in isolation.

However, when both branches were trained jointly under the multi-task HHA framework, the overall performance improved across all metrics. The Dice coefficient rose to 92.25%, while classification accuracy reached 99.53%, reflecting the synergistic interaction between the segmentation and classification pathways. This improvement illustrates that shared representation learning enables the network to leverage complementary cues as follows: segmentation benefits from class-level contextual guidance, while classification gains benefit from spatially aware feature embeddings produced by the decoder path.

These findings confirm that the proposed hybrid multi-task design effectively strengthens both segmentation precision and classification reliability through mutual information exchange, establishing its advantage over single-task counterparts.

### 4.5. Training Dynamics and Convergence Analysis

**Figure 7:**
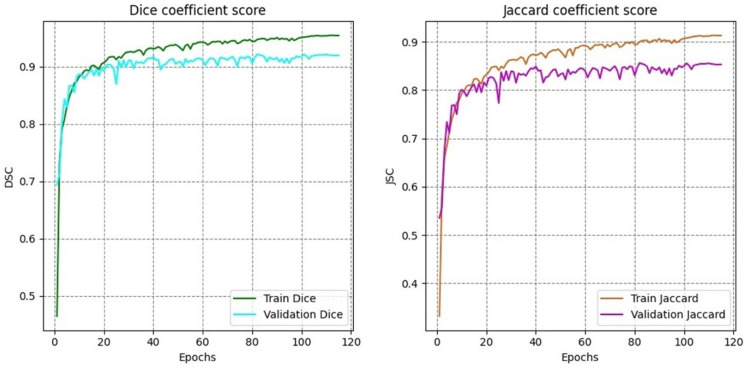
Training and validation curves for Dice coefficient and Jaccard index across epochs.

**Figure 8:**
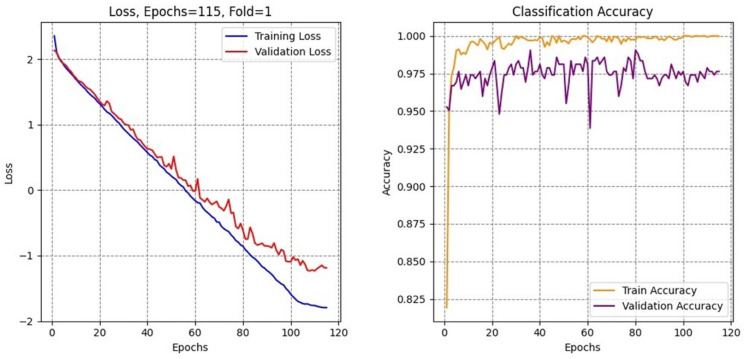
Curves of the training validation accuracy and loss.

The training curves for Dice coefficient and Jaccard index (Figure 7) reveal stable convergence behavior with minimal oscillation after the initial training phases. Both metrics show rapid improvement during the first thirty epochs, followed by gradual refinement throughout the remaining training. The close alignment between training and validation curves (Figure 8) indicates effective generalization without significant overfitting, attributable to the multi-task learning framework and robust regularization strategies.

### 4.6. Qualitative Results and Visual Analysis

**Figure 9:**
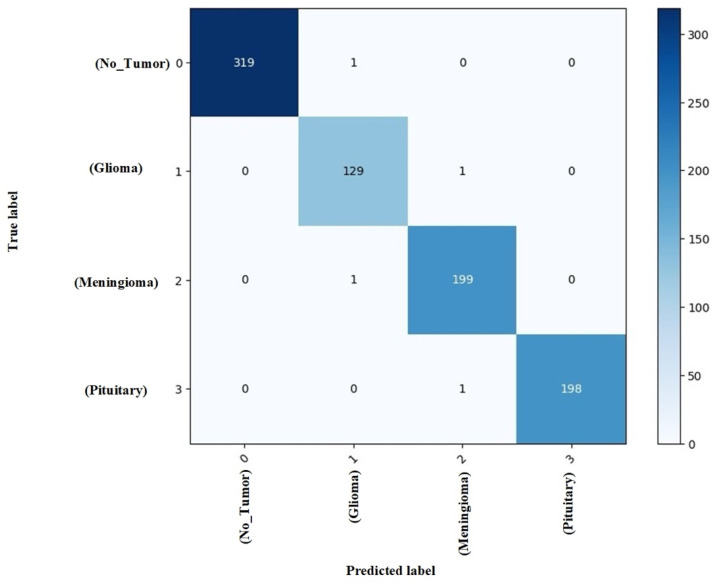
Confusion matrix analysis on the test set.

The confusion matrix (Figure 9) further confirms the model’s discriminative power, with minimal misclassification occurring predominantly between glioma and meningioma classes, which is consistent with known radiological challenges in differentiating these tumor types.

**Figure 10:**
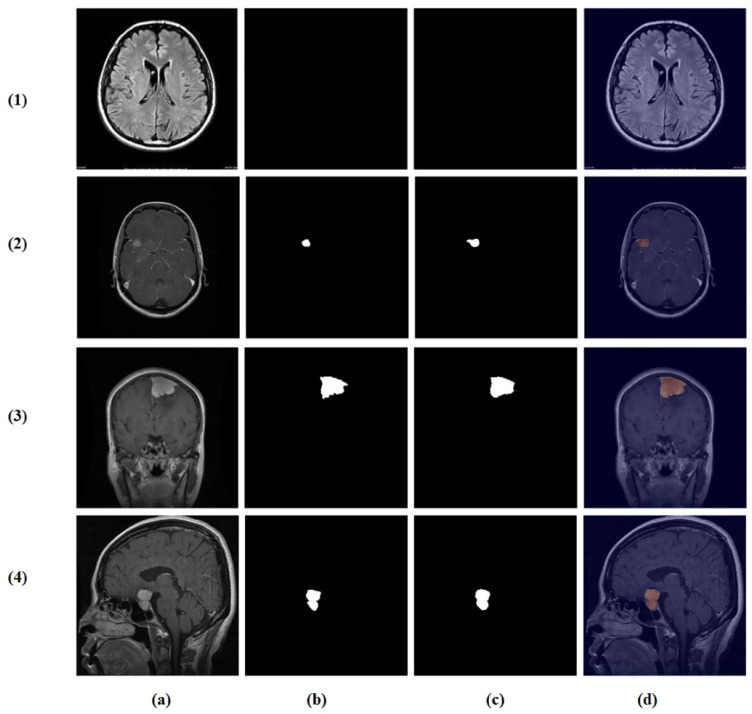
Random test set predictions. Columns: (**a**) input image, (**b**) ground truth mask, (**c**) predicted mask, and (**d**) overlay. Rows (true → predicted): (**1**) no tumor → no tumor, (**2**) glioma → glioma, (**3**) meningioma → meningioma, and (**4**) pituitary → pituitary.

Visual inspection of the segmentation outputs (Figure 10) demonstrates the model’s capability to accurately delineate tumor boundaries across diverse tumor types and imaging conditions. The model successfully handles variations in tumor size, shape, and location, producing precise segmentation masks that closely align with ground truth annotations.

**Figure 11:**
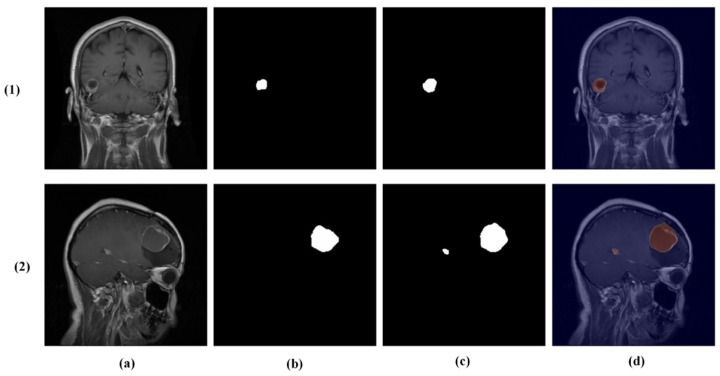
Challenging test set cases. Columns: (**a**) input image, (**b**) ground truth mask, (**c**) predicted mask, and (**d**) overlay. Rows (true → predicted): (**1**) glioma → meningioma (misclassification) and (**2**) pituitary tumor with under-segmentation.

While the model demonstrates strong overall performance, certain challenging cases remain difficult. As shown in Figure 11, these include misclassification between glioma and meningioma (row 1) and under-segmentation of tumors with faint boundaries (row 2). Such failures typically occur when tumors exhibit overlapping intensity profiles, low contrast with surrounding tissue, or ambiguous morphological features.

The simultaneous capability for accurate classification and precise segmentation addresses critical needs in neuro-oncological practice, where both tumor type identification and volumetric assessment are essential for treatment planning and monitoring. The model’s robustness across different data splitting strategies and its consistent performance across all tumor classes suggest strong generalizability.

The HHA mechanism effectively addresses key limitations of conventional U-Net architectures by selectively refining features during skip connections, suppressing noise while enhancing relevant structural information. The adaptive fusion gate dynamically balances global contextual understanding with local spatial details, enabling the network to handle the substantial heterogeneity inherent in brain tumor manifestations.

### 4.7. Feature Learning Analysis

**Figure 12:**
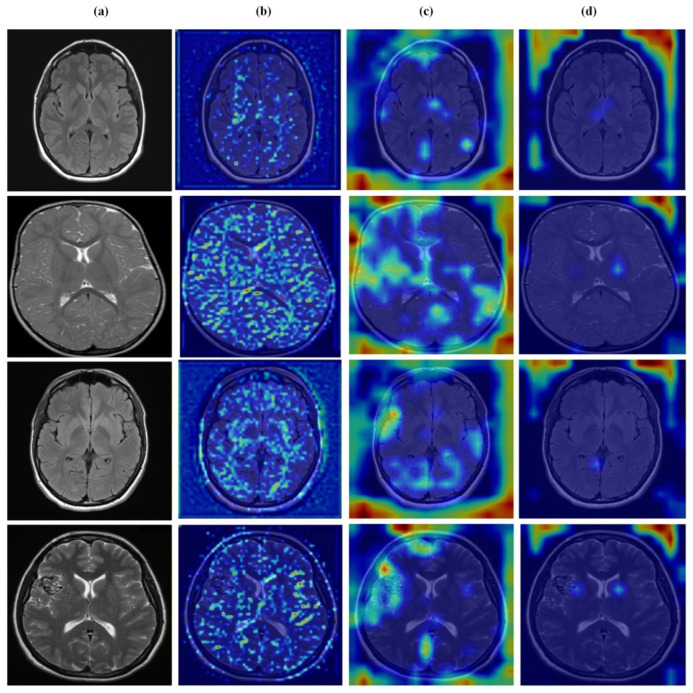
Visual representation of feature activations in no tumor cases. (**a**) input, (**b**) low-level features, (**c**) mid-level features, and (**d**) high-level features.

**Figure 13:**
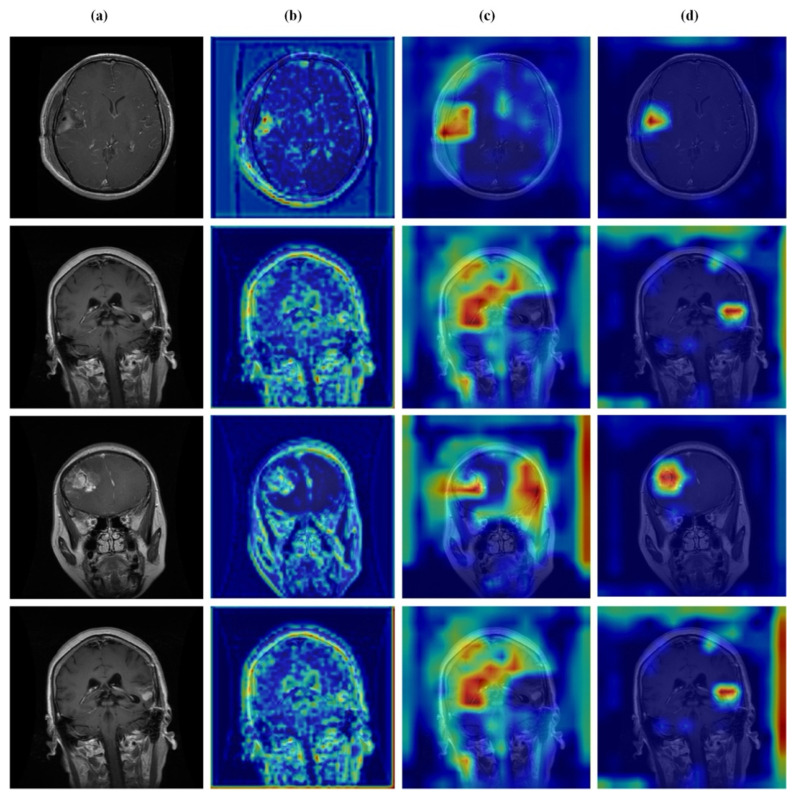
Visual representation of feature activations in glioma tumor cases. (**a**) input, (**b**) low-level features, (**c**) mid-level features, and (**d**) high-level features.

**Figure 14:**
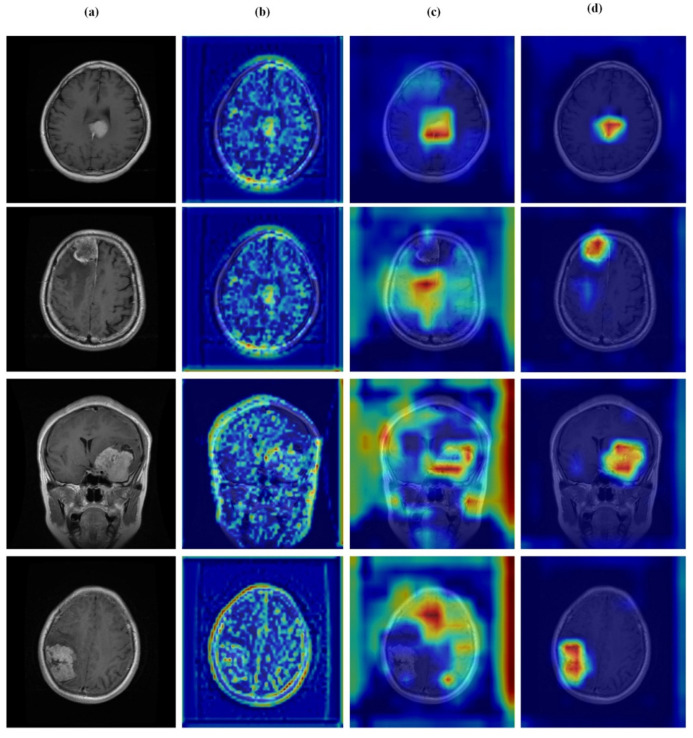
Visual representation of feature activations in meningioma tumor cases. (**a**) input, (**b**) low-level features, (**c**) mid-level features, and (**d**) high-level features.

**Figure 15:**
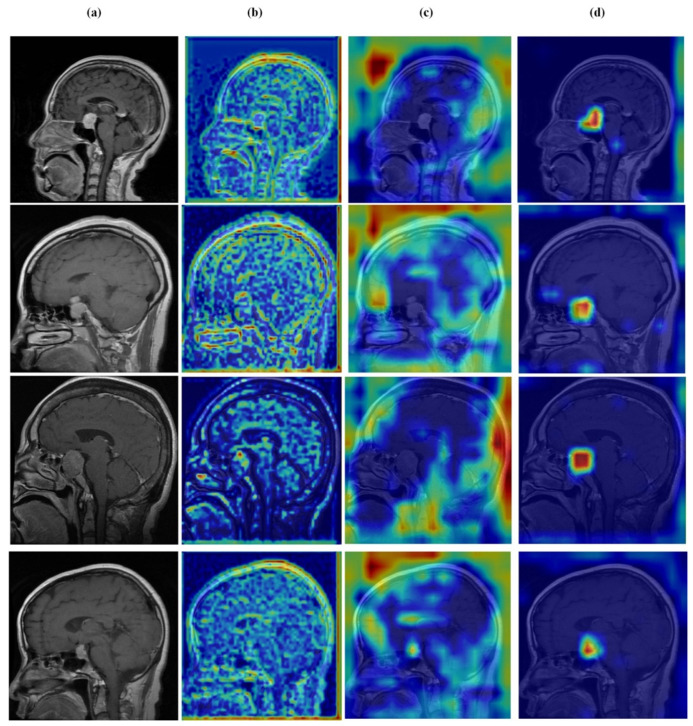
Visual representation of feature activations in pituitary tumor cases. (**a**) input, (**b**) low-level features, (**c**) mid-level features, and (**d**) high-level features.

Figure 12, Figure 13, Figure 14 and Figure 15 present Grad-CAM visualizations that trace the hierarchical feature learning of the proposed model across the four classes. Columns are organized as (a) input, (b) high-level features, (c) mid-level features, and (d) low-level features. In (d), the network emphasizes primitive cue edges, intensity transitions, and fine textures capturing sulcal boundaries, skull edges, and generic tissue patterns. Progressing to (c), activations become more structured and context-aware, highlighting coherent anatomical regions and diffuse hyperintense areas that provide spatial context for lesion localization. At (b), class-discriminative focus emerges with responses that contract tumor-centric hotspots for glioma and meningioma and to the sellar region for pituitary cases, while no_tumor images exhibit suppressed high-level responses, reflecting the model’s rejection of false lesion cues. This bottom-to-top progression from generic edges to task-specific evidence explains the model’s improved decision reliability and aligns with the multi-task design that encourages precise, clinically meaningful attention.

#### Limitation and Future Work

The proposed EfficientNetV2S HHA shows strong results, yet several constraints indicate directions for further study.

A central constraint arises from the training and evaluation data. The model was developed on the brain tumor segmentation dataset from Kaggle, which contains T1-weighted contrast-enhanced images drawn from a limited set of sources and likely specific imaging protocols. As a result, generalization to images from other hospitals, scanner vendors, magnetic field strengths, or different MRI sequences such as T2-weighted or FLAIR remains uncertain. Performance may decline under shifts in contrast, noise, or artifacts that are not represented in the training distribution.

The HHA adds computational cost. The dual branch design with adaptive fusion gates and multi-scale convolutional blocks increases parameters and inference time relative to standard U-Net models. This overhead may complicate use in real-time clinical settings or on resource-constrained hardware, including deployment at the point of care.

Interpretability also warrants attention. Although quantitative results are strong, the decision process within the attention modules is not fully transparent. Clear explanations of why specific regions are emphasized for classification or segmentation are important for clinical confidence and for diagnostic decision support, where explanatory rationale carries weight alongside predictive accuracy.

Future work should validate the model across diverse centers, vendors, field strengths, and MRI sequences; explore parameter efficient attention variants or pruning and quantization to reduce cost; and integrate attribution tools and uncertainty estimation to improve transparency and trust.

## 5. Conclusions

This paper introduced a multi-task framework built on EfficientNetV2S with Hierarchical Hybrid Attention (HHA) that performs brain tumor segmentation and classification within a single network. By transforming skip connections into selective regulators via correlation-driven fusion of global-context and local-spatial cues, the model delivers complementary gains across tasks as follows: 92.25% Dice and 86% Jaccard for segmentation, alongside 99.53% classification accuracy with near-perfect precision, recall, and F1. Ablations and comparisons with CBAM, ECA, and DSA confirm that the joint, per-pixel fusion strategy not merely stacking channel and spatial weights drives the improvement. Beyond raw scores, the framework’s design matters: multi-scale fusion consolidates deep semantics with fine detail, while the asymmetric heads exploit class-level cues to sharpen boundaries and, reciprocally, spatially aware features to stabilize recognition. The result is a compact, clinic-aligned pipeline that can map tumor extent and type in one pass.

## Figures and Tables

**Table 1:** Summary of key related works on brain tumor segmentation and classification.

Reference	Key Contribution	Identified Limitations
Taheri et al. [9]	Level set segmentation with a threshold-driven speed function.	Sensitive to noise and intensity inhomogeneity; struggles with low-contrast and diffuse tumor boundaries.
Islam et al. [10]	Combined superpixel generation with K-means clustering for tumor detection.	Relies on handcrafted features; performance limited by the clustering algorithm’s assumptions on tumor shape and texture.
Ronneberger et al. [11]	Introduced the U-Net architecture with symmetric encoder–decoder and skip connections for biomedical image segmentation.	Skip connections transfer encoder features directly, propagating noise and blurring boundaries; encoder capacity is often limited.
Oktay et al. [12]	Proposed Attention U-Net, using attention gates to selectively weigh features passed via skip connections.	Attention is applied sequentially (channel then spatial); does not explicitly model interactions between channel and spatial cues.
Zhang et al. [13]	Integrated attention gates into a Residual U-Net for brain tumor segmentation in MRI.	Inherits the axis-separable attention design; focuses solely on segmentation without joint classification.
Alom et al. [14]	Developed the Recurrent Residual U-Net (R2U-Net) to refine feature extraction through recurrent layers.	Increased computational complexity; may suffer from convergence issues or overfitting on smaller medical datasets.
Diakogiannis et al. [15]	Designed ResUNet-a, a deep learning framework with advanced residuals for semantic segmentation.	Originally tailored for remote sensing; may not fully capture the specific textures and contextual priors of brain tumors.
Zhao and Jia [16]	Employed a multi-scale CNN to handle the variable size of brain tumors.	A single-task model; lacks a mechanism for adaptive fusion of features from different scales.
Kamnitsas et al. [17]	Proposed DeepMedic, a dual-pathway 3D CNN utilizing multi-scale contextual information.	High computational and memory demands due to 3D processing; not designed for joint classification.
Rabby et al. [18]	Introduced BT-Net, a multi-task model based on VGG16 for segmentation, classification, and localization.	Uses a relatively weak VGG16 backbone; employs a simple attention mechanism without cross-feature interaction.
Kordnoori et al. [19]	Presented a deep multi-task model with a shared encoder for segmentation and classification.	Utilizes a standard CNN encoder without advanced attention; feature fusion is simplistic.
Hussain et al. [20]	Developed a Residual Attention U-Net for joint segmentation and classification.	Attention mechanism remains sequential and independent across channels and spatial dimensions.
Preetha et al. [21]	Combined a multi-scale Attention U-Net with an EfficientNetB4 encoder for brain tumor segmentation.	Focuses on a single task (segmentation); attention design does not jointly model global context and local spatial detail.

**Table 2:** Training and hyperparameters of the hybrid model.

Parameter Type	Value
Input image size	256 × 256 × 3
Batch size	16
Epoch	150
Optimizer	Adam
Learning rate (LR)	0.0001
Loss function	Multi-task: Dice Loss (segmentation) + Sparse Categorical Cross-Entropy (classification), weighted by AutomaticWeightedLoss
Dropout rates	0.3 (conv layers), 0.5 (dense layers)
Attention heads	4 (EnhancedHybridAttention)
Reduction ratio	8 (HybridDualAttention)

**Table 3:** Overall segmentation and classification performance of the proposed EfficientnetV2S HHA model on the test set.

Model	Dice (%)	Jaccard (%)	Accuracy (%)	Precision (%)	Recall (%)	F1_Score (%)
EfficientnetV2S HHA	92.25	85.77	99.53	99.53	99.48	99.53

**Table 4:** The classification report of proposed EfficientnetV2S HHA model obtained from the test set.

	Precision	Recall	F1_Score	Sensitivity	Specificity
NO_TUMOR	1.00	1.00	1.00	1.00	0.9981
GLIOMA_TUMOR	0.98	0.99	0.99	0.9923	0.9923
MENINGIOMA_TUMOR	0.99	0.99	0.99	0.98	0.9800
PITUITARY_TUMOR	1.00	0.99	1.00	1.00	0.9954

**Table 5:** Comparison of model performance across data split strategies.

Ratio of Splitting	Dice (%)	Jaccard (%)	Accuracy (%)	Precision (%)	Recall (%)	F1_Score (%)
80-10-10	91.99	85.36	98.82	98.84	99.03	98.82
**70-10-20**	**92.25**	**85.77**	**99.53**	**99.53**	**99.48**	**99.53**
70-15-15	92.00	85.46	99.21	99.22	98.89	99.21
60-20-20	91.29	84.17	98.82	98.82	98.59	98.82

**Table 6:** Ablation study results.

Model	Dice (%)	Jaccard (%)	Accuracy (%)	Precision (%)	Recall (%)	F1_Score (%)	Total Parameter	FLOPs (G)	Total Training Time
Attention U-Net	89.91	83.33	98.41	98.41	98.30	98.41	8,282,789	31.6	2 h 20 m 12 s
U-Net base model	89.63	82.67	98.58	98.61	98.47	98.58	28,503,781	108.7	3 h 2 m 48 s
EfficientNetV2S-base-model	90.13	83.56	96.35	96.40	96.32	96.36	39,443,858	150.5	3 h 47 m 47 s
EfficientNetV2S with EHA only	91.92	84.52	99.29	99.30	99.05	99.29	40,516,134	154.6	4 h 29 m 45 s
**EfficientNetV2S HHA**	**92.25**	**85.77**	**99.53**	**99.53**	**99.48**	**99.53**	55,247,068	210.8	4 h 8 m 29 s

**Table 7:** Segmentation performance comparison with state-of-the-art methods.

Author (Year)	Accuracy (%)	Dice (%)	Jaccard (%)
Ronneberger et al. (2015) [11]	88.03	83.45	84.2
Oktay et al. (2018) [12]	91.80	86.12	85.50
ZhenLiang et al. (2019) [28]	93.35	91.10	89.30
El-Shafai et al. (2022) [26]	93.47	35.18	21.34
Sobhaninia et al. (2020) [27]	-	80.03	-
Francisco et al. (2021) [29]	-	82.80	-
Mayala et al. (2022) [30]	-	84.69	74.43
Razzaghi et al. (2022) [31]	-	86.02	-
Sahoo et al. (2023) [32]	99.60	90.20	-
**The proposed EfficientNetV2S HHA**	**99.70**	**92.25**	**85.77**

**Table 8:** Classification accuracy comparison with state-of-the-art methods.

Author (Year)	Accuracy (%)
Ravinder et al. (2023) [33]	95.01
Cardoso et al. (2024) [34]	92.00
Ardan et al. (2024) [35]	95.00
Ullah et al. (2024) [36]	95.42
Syed et al. (2025) [20]	99.40
**The proposed EfficientNetV2S HHA**	**99.53**

**Table 9:** Comparison with state-of-the-art architectures on the test set.

Model Configuration	Dice (%)	Jaccard (%)	Accuracy (%)	Precision (%)	Recall (%)	F1 Score (%)
U-Net [11]	89.63	82.67	98.58	98.61	98.47	98.58
Attention U-Net [12]	89.91	83.33	98.41	98.41	98.30	98.41
R2U-Net [37]	77.97	64.78	96.46	96.46	95.78	96.45
U-Net3+ [38]	90.13	82.40	98.35	98.34	98.02	98.34
ResUnet-a [15]	88.21	80.76	96.81	97.00	95.07	96.75
Swin-Unet [39]	87.32	79.03	97.17	97.19	96.74	97.17
TransUnet [40]	90.25	83.05	98.88	98.90	98.45	98.87
**EfficientNetV2S HHA (the proposed model)**	**92.25**	**85.77**	**99.53**	**99.53**	**99.48**	**99.53**

**Table 10:** Performance of the proposed model across multiple runs with different random seeds.

	Dice (%)	Jaccard (%)	Accuracy (%)
Seed = 5	91.66	84.87	99.29
Seed = 40	91.29	84.25	99.20
Seed = 42	92.03	85.35	99.48
Seed = 32	91.21	84.11	99.43

**Table 11:** Comparison with different attention mechanisms.

Model Configuration	Dice (%)	Jaccard (%)	Accuracy (%)	Precision (%)	Recall (%)	F1 Score (%)
EfficientNetV2S + ECA [41]	89.41	83.12	97.06	97.18	96.93	97.05
EfficientNetV2S + DSA [42]	89.98	83.94	97.44	97.53	97.42	97.48
EfficientNetV2S + CBAM [43]	90.97	84.42	98.41	98.41	98.23	98.41
**EfficientNetV2S + HHA (the proposed model)**	**92.25**	**85.77**	**99.53**	**99.53**	**99.48**	**99.53**

**Table 12:** Comparison between segmentation, classification, and multi-task EfficientNetV2S HHA configurations.

Model Configuration	Dice (%)	Jaccard (%)	Accuracy (%)	Precision (%)	Recall (%)	F1_Score (%)
EfficientNetV2S HHA for Segmentation only	91.13	86.04	-	-	-	-
EfficientNetV2S HHA for Classification only	-	-	98.02	98.24	98.02	98.23
**EfficientNetV2S HHA**	**92.25**	**85.77**	**99.53**	**99.53**	**99.48**	**99.53**

## Data Availability

The data presented in this study are openly available in the ‘Brain Tumor Segmentation Dataset’ on Kaggle at https://www.kaggle.com/datasets/atikaakter11/brain-tumor-segmentation-dataset, accessed on 18 October 2025.
